# Social and economic capital as effect modifiers of the association between psychosocial stress and oral health

**DOI:** 10.1371/journal.pone.0286006

**Published:** 2023-05-18

**Authors:** Abby L. J. Hensel, Noha Gomaa

**Affiliations:** 1 Schulich School of Medicine and Dentistry, Western University, London, ON, Canada; 2 Epidemiology and Biostatistics, Schulich School of Medicine and Dentistry, Western University, London, ON, Canada; 3 Lawson Health Research Institute, London, ON, Canada; Universidad Cooperativa De Colombia - Pasto, COLOMBIA

## Abstract

**Objectives:**

To assess the extent of the association of psychosocial stress with oral health in an Ontario population stratified by age groups, and whether any association is modified by indicators of social and economic capital.

**Methods:**

We retrieved data of 21,320 Ontario adults, aged 30–74 years old, from the Canadian Community Health Survey (CCHS: 2017–2018), which is a Canada-wide, cross-sectional survey. Using binomial logistic regression models that adjusted for age, sex, education, and country of birth, we examined the association of psychosocial stress (indicated by perceived life stress) with inadequate oral health (indicated as having at least one of the following: bleeding gums, fair/poor self-perceived oral health, persistent oral pain). We assessed the effect measure modification of indicators of social (sense of belonging to the local community, living/family arrangements) and economic capital (household income, dental insurance, dwelling ownership) on the perceived life stress-oral health relationship, stratified by age (30–44, 45–59, 60–74 yrs). We then calculated the Relative Excess Risk due to Interaction (RERI) which indicates the risk that is above what would be expected if the combination of low capital (social or economic) and high psychosocial stress was entirely additive.

**Results:**

Respondents with higher perceived life stress were at a significantly higher risk of having inadequate oral health (PR = 1.39; 95% CI: 1.34, 1.44). Adults with low social and economic capital were also at an increased risk of inadequate oral health. Effect measure modification showed an additive effect of indicators of social capital on the perceived life stress-oral health relationship. This effect was evident across all three age groups (30–44, 45–59, 60–74 yrs), with the highest attributable proportion of social and economic capital indicators in the psychosocial stress-oral health relationship in older adults (60–74 yrs).

**Conclusion:**

Our findings suggest an exacerbating effect for low social and economic capital in the relationship of perceived life stress with inadequate oral health among older adults.

## Introduction

Oral diseases continue to constitute a significant burden to individuals and societies. In addition to impacting the quality of life, oral diseases are strongly linked to major health conditions such as cardiovascular diseases and diabetes and have been shown to have cumbersome direct and indirect costs to societies and health care systems [1]. This burden is inequitably distributed amongst social groups of the population creating pervasive oral health inequalities. Over the past two decades, there has been a surge in accumulating research investigating various aspects of oral health inequalities, including their fundamental causes, the pathways involved in their production, and their impact at the individual and societal levels.

Psychosocial factors have been extensively investigated in relation to oral health and health inequalities [2–5]. Several studies have shown that the prolonged exposure to chronic psychosocial stress is associated with an increased risk of poor oral health in both children and adults [6]. Psychosocial stress has also been found to be associated with the development and progression of oral and systemic diseases and to partly explain the social inequalities in oral and general health outcomes [7–9]. Meanwhile, positive psychosocial exposures such as social support and perceptions of self-efficacy and mastery are known to contribute to better oral health outcomes, such as having more functional teeth and enhanced oral health-related quality of life [7]. A number of pathways have been shown to link psychosocial factors to oral health, including the behavioural and biological components of the stress pathway. For example, individuals exposed to chronic stress are more likely to engage in health-harming behaviours such as smoking and alcohol, potentially as coping mechanisms. In addition, psychosocial stress elicits a biological stress response which results in a cascade of pathobiological alterations leading to inflammation and subsequent oral diseases [5, 10].

Meanwhile, theories from social epidemiology suggest that the relative social and economic positions of individuals and populations determine the magnitude of their exposure to psychosocial stress and its mitigation. The Commission on the Social Determinants of Health Framework [11] posits social and economic factors, such as income and education as well as the social support and social networks in which individuals are embedded on a daily basis, as fundamental causes of ill-health with psychosocial stress being as a strongly suggested pathway. Despite the large body of evidence on the links between structural, social, and economic factors with oral health, there appears to be little investigation on their possible buffering role on the impact of psychosocial stress on oral health. Investigating this is important for informing effective policies that aim to target oral health and oral health inequalities.

Social capital was described by Bordieu [12] and Coleman [13] as the social resources that are available to both an individual and the community through their social relationships that are characterized by mutual trust, such as social networks. Economic capital, on the other hand, refers to the material assets or resources that could be used to acquire or maintain better health, such as household income and health insurance [12, 14]. Both social and economic capital can influence health outcomes in various ways. Social capital can protect and strengthen health by encouraging engagement in healthy behaviours, reinforcing psychological resources such as self-esteem, supplying emotional support, and decreasing exposure to stressors [15]. Meanwhile, the impact of economic capital on health outcomes is evident in the health inequalities observed with the inequitable distribution of material resources. These in turn can determine the probability of an individual encountering a health problem or experiencing financial stress [14].

The relationship between social capital and oral health at both the community and individual level have been well-documented, where higher social capital such as having strong social relationships have been shown to have a protective effect on oral health. In adults, social support has been suggested to be a determinant of increased oral health-related quality of life [16], and good social capital has been shown to be associated with a lower prevalence of root decay [17]. On the other hand, loneliness and social isolation have been consistently linked to the onset of periodontal disease [18, 19]. Among older adults, low social capital has been shown to be associated with having fewer teeth [20] and increased periodontal attachment loss [21]. The relationship between economic capital and oral health is also well-investigated, and strongly supported. Most oral health outcomes such as oral health-related quality of life, dental caries and periodontal disease concentrate in individuals with lower household income, lower education, and those who lack dental insurance [22–27]. In adult clinical studies, higher income was shown to reduce the risk of periodontal disease [28], while the lack of financial support was associated with an increased number of missing and decayed teeth, as well as prevalence of oral pain in a Canadian population [24].

While there is a general consensus on the contribution of social and economic capital to oral health, their role in the relationship between psychosocial stress and oral health is less understood. We derive our hypotheses based on the *buffering hypothesis* described by Cohen and Wills [29] which postulates that the effect of psychosocial stress on health outcomes may be *buffered*, i.e. modified by levels of social support [30], and which we expand on by investigating the *buffering* or modifying role of social capital. As well, we build on previous research showing economic factors to modify the relationship between stress and health outcomes. Our objectives in this study were to quantify the extent to which psychosocial stress, measured using perceived life stress, is associated with oral health, and then determine the extent to which indicators of social (sense of belonging to the local community and living/family arrangements) and economic capital (household income, dental insurance, and dwelling ownership) can modify this relationship. Our hypothesis was that in lower social and economic capital individuals, there will be a stronger association between psychosocial stress and oral health.

## Methods

### Data source and study population

We used data from the Canadian Community Health Survey (CCHS) from the 2017–2018 cycle. CCHS is a cross-sectional survey conducted by Statistics Canada that collects information related to health status, health care utilization, and health determinants among Canadians aged 12 and older [31]. The survey excluded residents of Indian reserves, health care institutions, some remote areas, and full-time members of the Canadian Forces [31]. Data were collected using the CCHS questionnaire designed for computer assisted interviewing [31]. Both telephone and online electronic interviews were used to collect data, and each interview had an entry component, a health content, and an exit component. Each group of questions in the CCHS focused on a particular theme of health, such as general health, chronic conditions, smoking, and alcohol use. Some modules, such as dental visits and oral health, were part of the 2017–2018 cycle’s optional content. Therefore, our analysis focused on adults in Ontario, Canada’s most populated province, between the ages of 30 and 74 years old with complete data, for a total sample of 21,320, which represents a weighted sample of approximately 8,175,534 Ontario residents.

### Variables

#### Exposure

*Psychosocial stress*. Psychosocial stress was measured in CCHS using single-item Likert responses to the question “Thinking about the amount of stress in your life, would you say that most days are … not at all stressful, not very stressful, a bit stressful, quite a bit stressful, or extremely stressful?” The reliability and validity of single-item psychosocial stress measures compared to multiple item instruments has been established, showing their usefulness in large population-based epidemiological studies [32]. Given the ordinal level of measurement, we dichotomized this variable as “low stress” (*not at all stressful*, *not very stressful*, *a bit stressful*) and “high stress” (*quite a bit stressful*, *extremely stressful*).

#### Effect modifiers

*Social capital*. Indicators of social capital included two variables: sense of belonging to the local community, living/family arrangements, and household size. Sense of belonging to the local community, originally measured in CCHS on a 4-point scale from very strong to very weak, was dichotomized as “low sense of belonging” (very weak, somewhat weak) and “high sense of belonging” (very strong, somewhat strong). Living/family arrangements was characterized using a series of eight variables: unattached individual living alone; unattached individual living with others; individual living with spouse/partner; parent living with spouse/partner and child(ren); single parent living with children; child living with a single parent with or without siblings; child living with two parents with or without siblings; and other. We further dichotomized living/family arrangements as “living alone” (unattached individual living alone) and “living with others”.

*Economic capital*. Indicators of economic capital included three variables: total household income, having dental insurance and dwelling ownership. In Ontario, the low-income cut-off is determined based on family size (i.e., number of persons living in one household) and community size (i.e., number of persons living in a community) [33]. In 2017 and 2018, a family size of three persons and a community size of 500,000 persons or more had a low-income cut-off of $31,822 and $32,554, respectively [34]. Therefore, since more than 80% of our sample had three persons or less per home, we dichotomized household income as “low income” (less than $39,999) and “middle/high” (more than $40,000). Whether a respondent had dental insurance was dichotomized as “yes” and “no. Finally, dwelling ownership was dichotomized in CCHS as “owned by member of household” and “rented”.

#### Outcome

*Oral health*. Inadequate oral health was indicated as having at least one of the following oral health outcomes: bleeding gums, fair/poor perceived oral health and persistent oral pain. Bleeding gums was characterized in CCHS as the frequency of bleeding gums in the previous 12 months using the following four items: often; sometimes; rarely, and never. We further dichotomized bleeding gums as “present” (often, sometimes) and “not present” (rarely, never). Perceived oral health was measured in CCHS on a 5-point Likert scale from poor to excellent that asked the respondents to self-rate their oral health. We dichotomized perceived oral health as “fair/poor” (poor, fair) and “good” (good, very good, excellent). Finally, persistent oral pain was characterized as the frequency of persistent pain related to problems with the mouth in the previous 12 months using the following four items: often; sometimes; rarely; and never, and was further dichotomized as “present” (often, sometimes) and “not present” (rarely, never).

#### Covariates

We accounted for confounders that were selected *a priori* based on the literature suggesting these as potentially associated with both exposure and outcome. These included age (categorized as provided in CCHS into 30–44 years; 45–59 years and 60–74 years old,), biological sex (male, female), level of education (less than secondary school, secondary school and post-secondary education) and country of birth (Canada, other), as a proxy for immigration status.

### Statistical analyses

First, we applied descriptive statistics to assess the characteristics of our study population using chi-squared tests. Next, we constructed multivariable binomial logistic regression models to estimate the prevalence ratios (PR) and 95% confidence intervals (CI) that quantify the association between perceived life stress (exposure) and inadequate oral health (binary outcome), adjusted for age, sex, level of education and country of birth.

We then followed the methods described by Knol and VanderWeele [35] to assess the effect measure modification (EMM) of indicators of social and economic capital on the association between perceived life stress and inadequate oral health, stratified by age group and adjusted for confounders. We estimated the relative excess risk due to interaction (RERI) as a standard measure for EMM on the additive scale. RERI corresponds to the risk that is in excess of what would be expected if the combination of psychosocial stress and social/economic capital was entirely additive. The RERI was calculated using the following equation: RERI = PR_11_ –PR_10_ –PR_01_ + 1, where PR_*ab*_ is the prevalence risk (PR) in the group with *X*_1_ exposure status *a* (1 = exposed; 0 = unexposed) and *X*_2_ exposure status *b* (1 = exposed; 0 = unexposed). The RERI was interpreted according to its direction, as opposed to its size. A positive value suggests that the combination of both exposures is higher than the sum of their independent effects on oral health.

The attributable proportion (AP) was also estimated. This measure was used to capture the proportion of the disease in the doubly exposed group that is due to the interaction between the two exposures [36]. The AP was calculated using the following equation: AP = RERI / PR_11_, where PR_11_ is the prevalence risk in the group with exposure to both *X*_1_ and *X*_2_. The Results of the EMM analyses are presented as proposed by Knol and VanderWeele [35]. All analyses were conducted using Stata/BE 17.0 (College Station, Texas).

### Ethical considerations

As this was an analysis of publicly available data, we did not require approval from the Research Ethics Board at Western University, according to the Tricouncil policy statement for ethical conduct for research involving humans. No informed consent was required. Statistics Canada anonymized all registered data before the data were made available on their secured server.

## Results

### Characteristics of study population

A total of 21,320 participants were included in the analysis of whom 44.9% (n = 9,584) were men, with a mean age (± standard deviation) of 51.2 (±10.19). Characteristics of respondents included in this study are described in Table 1. Inadequate oral health (having at least one of the oral health outcomes: bleeding gums, fair/poor perceived oral health, and persistent oral pain) was equally distributed between both sexes and was most prevalent in the younger age group (30–44 years old, 43.8%). A greater proportion of participants who reported experiencing higher levels of perceived life stress also had inadequate oral health outcomes (47.0%) compared to those who reported low perceived life stress (32.7%). Men and women who reported experiencing higher levels of perceived life stress had higher prevalence of inadequate oral health (26.6% and 31.5%, respectively) compared to those who reported low perceived life stress (17.5% and 19.4%, respectively). Participants who had a low sense of belonging to the local community, a total household income below $40,000 per annum, and who rented their dwelling also had a higher prevalence of inadequate oral health (44.4%, 46.4%, and 47.6%, respectively).

**Table 1:** Descriptive statistics of sample by biological sex and oral health status (“inadequate” and “adequate”), n = 21,320: Canadian Community Health Survey (2017–2018).

Total	N = 8,175,534	Inadequate Oral Health		*P*-value*	Adequate Oral Health		*P*-value*
		Female	Male		Female	Male	
	n = 21,320	n = 4,266	n = 3,403		n = 7,470	n = 6,181	
	100%	55.60%	44.40%		54.70%	45.30%	
***Sociodemographic characteristics***							
**Age (years)**							
30–44	37.8	44.6	42.9	0.058	36.5	37.4	0.472
45–59	37	36.6	39.3		37.2	37.1	
60–74	25.2	18.8	17.8		26.3	25.5	
**Education**							
Less than high school	6.62	6.8	8.7	0.003	4.3	5.2	0.056
High school	16.76	17.1	17.8		16.1	15.6	
Post-secondary/ college/university	76.62	76.1	73.4		79.6	79.2	
**Marital status**							
Partner	62.3	56.8	62.1	<0.001	61.7	32.4	<0.001
No partner	37.7	43.2	37.9		38.3	67.6	
**Country of birth**							
Canada	77	75.7	76.7	0.335	77.6	77.4	0.761
Other	23	24.3	23.3		22.4	22.6	
***Psychosocial stress***							
**Perceived life stress**							
Not at all stressful/not very stressful/a bit stressful	77.8	68.5	73.4	<0.001	80.6	82.5	0.005
Quite a bit stressful/extremely stressful	22.2	31.5	26.6		19.4	17.5	
***Social capital factors***							
**Sense of belonging to local community**							
High sense of belonging	70.9	64.4	63.4	0.373	76	73.3	<0.001
Low sense of belonging	29.1	35.6	36.6		24	26.7	
**Living/family arrangements**							
Alone	30.2	30.6	32.6	0.066	29.2	29.4	0.816
With others	69.8	69.4	67.4		70.8	70.6	
***Economic capital factors***							
**Household income, $**							
<39,999	22.1	29.1	25	<0.001	19.6	15.5	<0.001
>40,000	77.9	70.9	75		80.4	84.5	
**Dental insurance**							
Yes	65.9	65.4	64.8	0.542	67	68.9	0.017
No	34.1	34.6	35.2		33	31.1	
**Home ownership**							
Owns	76.9	69.4	71.6	0.033	81.2	82.1	0.189
Rents	23.1	30.6	28.4		18.8	17.9	
***Oral health factors***							
**Perceived oral health**							
Good	87.3	67.5	57.2	<0.001			
Poor	12.7	32.5	42.8				
**Bleeding gums**							
Often/sometimes	23	66.4	64.5	0.092			
Rarely/never	77	33.6	35.5				
**Persistent oral pain**							
Often/sometimes	12.5	40.5	30.9	<0.001			
Rarely/never	87.5	59.5	69.1				

*p-value results for Chi-square test.

### *Association* between psychosocial stress and inadequate oral health outcomes

We used multivariable binomial logistic regression models to assess the association between perceived life stress and inadequate oral health. Our results showed individuals who reported having high levels of perceived life stress to be at a greater risk of experiencing inadequate oral health (PR = 1.39; 95% CI = 1.34, 1.44) than those who reported low perceived life stress.

### Effect of social capital on the association of psychosocial stress with inadequate oral health across age groups

Living/family arrangements modified the association between perceived life stress and inadequate oral health across all three age groups, while sense of belonging to the local community only modified this relationship in adults aged 45–59 and 60–74 years old (Table 2). In younger adults (30–44 years old), low sense of belonging and high perceived life stress had a 1.57 (95% CI: 1.46, 1.69) higher risk of inadequate oral health compared to respondents with high sense of belonging and low perceived life stress (reference group); however, sense of belonging to the local community in this age group did not contribute to the stress-oral health relationship (RERI = -0.04). Younger adults who lived alone and have high perceived life stress also had a higher risk of inadequate oral health (PR = 1.33; 95% CI: 1.21, 1.47) and contributed to 6.0% of the association between stress and oral health (RERI = 0.08).

**Table 2:** Effect measure modification of indicators of social capital on the association between perceived life stress and inadequate oral health (bleeding gums, fair/poor perceived oral health, and persistent oral pain) stratified by age (30–44, 45–59, 60–74 years old): Canadian Community Health Survey (2017–2018).

	Low perceived life stress		High perceived life stress		PR (95% CI) perceived life stress within strata of social capital
	N with/without inadequate oral health	PR (95% CI)	N with/without inadequate oral health	PR (95% CI)	
**30–44 years old (N = 8,587)**					
*Sense of belonging to the local community*					
High sense of belonging	1,442/2,794	1.0 (Ref)	623/784	1.31	1.31
				(1.21, 1.41)	(1.22, 1.41)
Low sense of belonging	793/1,000	1.3	462/407	1.57	1.2
		(1.22, 1.39)		(1.46, 1.69)	(1.11, 1.31)
RERI (additive scale): 1.57–1.31–1.30 + 1 = -0.04					
Attributable proportion: N/A					
*Living Arrangements*					
Living with others	1,628/2,791	1.0 (Ref)	769/878	1.28	1.28
				(1.20, 1.36)	(1.20, 1.36)
Living alone	444/765	0.97	237/228	1.33	1.38
		(0.89, 1.05)		(1.21, 1.47)	(1.22, 1.55)
RERI (additive scale): 1.33–1.28–0.97 + 1 = 0.08					
Attributable proportion: 0.08/1.33 = 6.0%					
**45–59 years old (N = 8,412)**					
*Sense of belonging to the local community*					
High sense of belonging	1,335/2,997	1.0 (Ref)	448/664	1.31	1.31
				(1.20, 1.43)	(1.21, 1.43)
Low sense of belonging	630/987	1.26	421/339	1.79	1.42
		(1.17, 1.36)		(1.66, 1.94)	(1.30, 1.55)
RERI (additive scale): 1.79–1.31–1.26 + 1 = 0.22					
Attributable proportion: 0.22/1.79 = 12.3%					
*Living Arrangements*					
Living with others	1,215/2,583	1.0 (Ref)	492/636	1.37	1.37
				(1.26, 1.49)	(1.26, 1.49)
Living alone	665/1,268	1.05	346/338	1.54	1.47
		(0.97, 1.13)		(1.41, 1.68)	(1.33, 1.62)
RERI (additive scale): 1.54–1.37–1.05 + 1 = 0.12					
Attributable proportion: 0.12/1.54 = 7.8%					
**60–74 years old (N = 5,730)**					
*Sense of belonging to the local community*					
High sense of belonging	818/2,587	1.0 (Ref)	149/226	1.65	1.64
				(1.43, 1.89)	(1.43, 1.89)
Low sense of belonging	297/583	1.4	107/78	2.36	1.69
		(1.25, 1.89)		(2.06, 2.71)	(1.45, 1.98)
RERI (additive scale): 2.36–1.65–1.40 + 1 = 0.71					
Attributable proportion: 0.71/2.36 = 30.1%					
*Living Arrangements*					
Living with others	649/2,043	1.0 (Ref)	135/190	1.71	1.69
				(1.47, 1.99)	(1.46, 1.96)
Living alone	424/1,072	1.14	115/97	2.1	1.85
		(1.02, 1.27)		(1.81, 2.44)	(1.59, 2.15)
RERI (additive scale): 2.10–1.71–1.14 + 1 = 0.25					
Attributable proportion: 0.25/2.10 = 12.5%					

Note: All estimates adjusted for sex, education, and country of birth.

Middle-aged adults (45–59 years old) who had high perceived stress and low sense of belonging to the local community (PR = 1.79; 95% CI: 1.66, 1.94) or who lived alone (PR = 1.54; 95% CI: 1.41, 1.68) continued to have a higher risk of inadequate oral health, and contributed to 12.3% (RERI = 0.22) and 7.8% (RERI = 0.12) of the perceived life stress and oral health association, respectively.

Both indicators of social capital had the largest magnitude of effect modification in older adults (60–74 years old). Older adults who had high perceived life stress and low sense of belonging to the local community (PR = 2.36; 95% CI: 2.06, 2.71) or high perceived life stress and who lived alone (PR = 2.10; 95% CI: 1.81, 2.44) had a higher risk of inadequate oral health and contributed to 30.1% (RERI = 0.71) and 12.5% (RERI = 0.25) of the association between perceived life stress and oral health, respectively.

### Effect of economic capital on the association of psychosocial stress with inadequate oral health across age groups

Indicators of economic capital also modified the association between psychosocial stress and inadequate oral health (Table 3). The magnitude of effect was highest in older adults (60–74 years old) for all three indicators.

**Table 3:** Effect measure modification of indicators of economic capital on the association between perceived life stress and inadequate oral health outcomes (at least one of bleeding gums, fair/poor perceived oral health, and persistent oral pain) stratified by age (30–44, 45–59, 60–74 years old): Canadian Community Health Survey (2017–2018).

	Low perceived life stress		High perceived life stress		PR (95% CI) for perceived life stress within strata of social capital
	N with/without inadequate oral health	PR (95% CI)	N with/without inadequate oral health	PR (95% CI)	
**30–44 years old (N = 8,587)**					
*Household income*					
$40,000 or more	1,753/3,298	1.0 (Ref)	781/993	1.28	1.28
				(1.20, 1.37)	(1.20, 1.37)
$39,999 or less	500/533	1.31	316/202	1.65	1.25
		(1.21, 1.42)		(1.52, 1.79)	(1.14, 1.38)
RERI (additive scale): 1.65–1.28–1.31 +1 = 0.06					
Attributable proportion: 0.06/1.65 = 3.6%					
*Dental insurance*					
Yes	1,656/2,982	1.0 (Ref)	795/934	1.29	1.29
				(1.21, 1.38)	(1.21, 1.38)
No	589/848	1.1	299/261	1.45	1.31
		(1.02, 1.19)		(1.33, 1.58)	(1.18, 1.44)
RERI (additive scale): 1.45–1.29–1.10 + 1 = 0.06					
Attributable proportion: 0.06/1.45 = 4.1%					
*Home ownership*					
Owns	1,501/2,885	1.0 (Ref)	667/892	1.25	1.26
				(1.17, 1.34)	(1.17, 1.35)
Rents	727/894	1.3	411/287	1.74	1.31
		(1.22, 1.40)		(1.61, 1.87)	(1.20, 1.42)
RERI (additive scale): 1.74–1.25–1.30 + 1 = 0.19					
Attributable proportion: 0.19/1.74 = 10.9%					
**45–59 years old (N = 8,412)**					
*Household income*					
$40,000 or more	1,488/3,315	1.0 (Ref)	586/828	1.34	1.34
				(1.24, 1.45)	(1.24, 1.45)
$39,999 or less	505/714	1.31	293/185	1.94	1.49
		(1.20, 1.42)		(1.78, 2.12)	(1.35, 1.64)
RERI (additive scale): 1.94–1.34–1.31 +1 = 0.29					
Attributable proportion: 0.29/1.94 = 14.9%					
*Dental insurance*					
Yes	1,331/2,898	1.0 (Ref)	625/749	1.45	1.45
				(1.35, 1.57)	(1.35, 1.56)
No	665/1,129	1.16	248/260	1.5	1.3
		(1.08, 1.26)		(1.35, 1.66)	(1.17, 1.45)
RERI (additive scale): 1.50–1.45–1.16 + 1 = -0.11					
Attributable proportion: N/A					
*Home ownership*					
Owns	1,514/3,409	1.0 (Ref)	580/818	1.35	1.36
				(1.25, 1.46)	(1.26, 1.47)
Rents	462/580	1.43	288/185	1.98	1.34
		(1.33, 1.56)		(1.82, 2.16)	(1.21, 1.48)
RERI (additive scale): 1.98–1.35–1.43 + 1 = 0.20					
Attributable proportion: 0.20/1.98 = 10.1%					
**60–74 years old (N = 5,730)**					
*Household income*					
$40,000 or more	786/2,514	1.0 (Ref)	152/238	1.63	1.63
				(1.41, 1.87)	(1.41, 1.88)
$39,999 or less	349/699	1.34	109/75	2.33	1.75
		(1.20, 1.50)		(2.02, 2.69)	(1.50, 2.03)
RERI (additive scale): 2.33–1.63–1.34 +1 = 0.36					
Attributable proportion: 0.36/2.33 = 15.5%					
*Dental insurance*					
Yes	453/1,517	1.0 (Ref)	96/139	1.73	1.77
				(1.45, 2.08)	(1.48, 2.11)
No	679/1,688	1.21	164/174	2.03	1.68
		(1.09, 1.34)		(1.76, 2.33)	(1.48, 1.91)
RERI (additive scale): 2.03–1.73–1.21 + 1 = 0.09					
Attributable proportion: 0.09/2.03 = 4.4%					
*Home ownership*					
Owns	886/2,720	1.0 (Ref)	165/247	1.61	1.58
				(1.41, 1.84)	(1.38, 1.81)
Rents	242/463	1.4	93/61	2.45	1.8
		(1.24, 1.57)		(2.13, 2.82)	(1.53, 2.11)
RERI (additive scale): 2.45–1.61–1.40 + 1 = 0.44					
Attributable proportion: 0.44/2.45 = 17.9%					

Note: All estimates adjusted for sex, education, and country of birth.

Across all three age groups, respondents who had high life stress and low household income (<$40,000) had a higher risk of inadequate oral health. The magnitude of effect modification was highest among older adults (60–74 years old; PR = 2.33; 95% CI: 2.02, 2.69), contributing to 15.5% (RERI = 0.36) of the association between psychosocial stress and oral health. Household income contributed to 3.6% (RERI = 0.06) and 14.9% (RERI = 0.29) of the stress-oral health relationship in younger adults (30–44 years old) and middle-aged adults (45–59 years old), respectively.

Younger adults who had high perceived life stress and no dental insurance had a 1.45 (95% CI: 1.33, 1.58) higher risk of inadequate oral health compared to respondents with low perceived life stress who had dental insurance (reference group). Middle-aged (PR = 1.50; 95% CI: 1.35, 1.66) and older adults (PR = 2.03; 95% CI: 1.76, 2.33) also had a higher risk of inadequate oral health. Dental insurance had an attributable proportion of 4.1% (RERI = 0.06) and 4.4% (RERI = 0.09) for younger adults and older adults, respectively. Dental insurance did not contribute to the association between psychosocial stress and oral health in middle-aged adults (RERI = -0.11).

Finally, homeownership modified 10.9% (RERI = 0.19), 10.1% (RERI = 0.20), and 17.9% (RERI = 0.44) of the psychosocial stress and oral health relationship in younger, middle-aged, and older adults, respectively. Older adults who reported having high perceived life stress and who rented their home had a 2.45 (95% CI: 2.13, 2.82) higher risk of inadequate oral health, while younger adults and middle-aged adults had a 1.74 (95% CI: 1.61, 1.87) and 1.98 (95% CI: 1.82, 2.16) higher risk of inadequate oral health, respectively.

## Discussion

In our study, we used methods of EMM to assess the extent to which indicators of social and economic capital modified the association between psychosocial stress and inadequate oral health in a large, provincially representative sample of adults from Ontario—Canada’s most populated province. Our findings indicate that psychosocial stress, indicated by perceived life stress, is significantly associated with inadequate oral health, supporting previous studies with similar findings [7, 9, 37].

We also demonstrated that social and economic capital are modifiers of the psychosocial stress-oral health relationship, where better social and economic capital can be protective for oral health against effects of psychosocial stress, and that the magnitude of effect modification increased with age. It is well-known established that oral health declines with age [38]. For example, individuals aged 60 years and older have the highest incidence of dental caries, periodontal disease, oral cancer, and edentulism [39]. Economic capital has also been shown to decrease over time, where financial resources decline across the lifespan, particularly after retirement [40]. However, the literature is limited as to whether social capital follows a similar pattern. Social isolation and loneliness have been identified as risk factors for poor mental and physical health, particularly in older adults, and have been linked to reduced social networks, decreased economic resources, and changes in family structure and mobility [41]. Alternatively, other research suggests that older individuals experience an increase in neighbourhood socializing and volunteering, demonstrating flexibility and adaptability in maintaining friendship networks over time [42, 43]. Our results suggest that social and economic capital may decrease with age, exacerbating the effects of psychosocial stress on oral health as individuals get older.

Epidemiological theories of the social production of disease propose that social and economic positioning determine an individual’s exposure to health-damaging stressors and allow for an improved access to various resources that aid in avoiding risks and mitigating the impact of the disease generation process [37]. For example, social capital at the individual level may affect oral health through social norms and informational social control, as well as through the diffusion of health-related knowledge through social networks [44, 45]. Our findings are in support of previous research demonstrating that social capital protects against poor oral health outcomes. For example, one study found that in older adults, low social capital was a predictor of poor oral health-related quality of life [44]. Another study found adults reporting small social networks to be at a higher risk of having poor self-rated oral health [46]. Therefore, when individuals experience psychosocial stress, it is possible that low social capital, such as the lack of social networks or social support, can exacerbate the impact of psychosocial stress and contribute to the development of poor oral health.

A biopsychosocial mechanism may also explain the modifying role of social capital. Poor social resources are postulated to increase stressful experiences that an individual might encounter as well as negatively impact health [47]. One way to characterize the biological response to chronic stress is by measuring allostatic load, which is a biological indicator of the “*wear and tear*” of physiological functioning resulting from the repeated and prolonged exposures to stressors [3, 47, 48]. Increased allostatic load has been demonstrated to increase the risks of several health conditions, including cardiovascular disease [48, 49], depression [50, 51], and periodontal disease [2, 10]. This adds to the biological plausibility that the impact of psychosocial stress on oral health can be mitigated in individuals with a higher social capital, such as increased social networks or sense of belonging to the local community which in turn may ameliorate the stressful exposures and curb the biological stress toll [47].

Previous research has demonstrated that the relationship between current stress and poor oral health may also be moderated by indicators of economic capital such as socioeconomic position and the availability of dental insurance [52]. Low economic capital may exacerbate a multitude of health barriers related to oral health. For example, individuals with low economic capital may experience: (1) worse access to adequate dental care due to unemployment or employment in a position with poor health benefits [53]; (2) greater chronic stress and stress-coping behaviours that are more damaging to oral health (e.g., tobacco use, alcohol use, sugar consumption) [52]; and/or (3) greater risk of other chronic diseases, which may impact oral disease via the oral-systemic health link [54–56]. Our findings that indicators of economic capital, such as household income, dental insurance, and dwelling ownership, modify the association between psychosocial stress and oral health support the hypothesis that individuals with higher economic capital can mitigate the possible impacts of psychosocial stress on poor oral health.

The findings of our study further highlight the need for initiatives that address the social determinants of health, particularly ones that tackle elements of social and economic capital in order to mitigate the harmful impact of stress. For example, strategies can be suggested to support community and local environments that enhance an individual’s sense of belonging and engagement in their community to promote their oral and general health. Similarly, policies that strengthen economic capital such as those directed towards enhancing income and housing equality and universal access to dental care can also protect against the negative impacts of psychosocial stress on oral health [26, 57, 58].

### Strengths and limitations

Some of the strengths of our study include our use of a large, provincially representative sample of adults aged 30–74 years old. Also, we used multiple indicators to generate a construct variable of oral health. We also used multiple indicators for each of our social and economic capital variables to ensure that our results are not sensitive to a specific measure, but rather applicable to various exposure variables. Our study also has a few limitations, including its reliance on self-reported measures of perceived life stress, social capital, economic capital, and oral health, which may make the responses subject to recall and social desirability biases [59]. Our study is also limited by the cross-sectional nature of CCHS data and subsequently our cross-sectional study design, which limits our ability to make inferences about temporal sequence or causation. In order to better understand the mechanisms linking psychosocial stress to oral health, and to gain further insights into the roles of social and economic capital in this relationship, longitudinal study designs and/or causal inference analyses can be employed in future studies.

## Conclusion

Overall, our findings corroborate the notion that perceived life stress increases the risk of inadequate oral health. We demonstrate that this relationship is buffered by enhanced social and economic capital. Within its limits, our study warrants greater attention to the role of psychosocial stress in oral diseases, and importantly, necessitates action on the social and economic factors that can play a role in mitigating the impact of stress on oral health. Future work should examine possible social, psychosocial, and biological mechanisms linking social and economic capital to inadequate oral health.

## References

[1] Jin LJ, Lamster IB, Greenspan JS, Pitts NB, Scully C, Warnakulasuriya S. Global burden of oral diseases: Emerging concepts, management and interplay with systemic health. Oral Diseases. 2015;22: 609–19. 10.1111/odi.12428 26704694

[2] Sabbah W, Watt RG, Sheiham A, Tsakos G. Effects of allostatic load on the social gradient in ischaemic heart disease and periodontal disease: Evidence from the third national health and nutrition examination survey. J Epidemiol Community Health. 2008;62(5): 415–20. doi: 10.1136/jech.2007.064188 18413454

[3] Gomaa N, Glogauer M, Nicolau B, Tenenbaum H, Siddiqi A, Fine N, et al. Stressed-out oral immunity: A gateway from socioeconomic adversity to periodontal disease. Psychosom. Med. 2020;82: 126–37. doi: 10.1097/PSY.0000000000000774 31860530

[4] Fagundes MLB, Júnior OLA, Menegazzo GR, Bastos LF, Hugo FN, Abreu LG, et al. Pathways of socioeconomic inequalities in self-perceived oral health. Braz Oral Res. 2022;36: e088. doi: 10.1590/1807-3107bor-2022.vol36.0088 35703713

[5] Park L, Gomaa N, Quiñonez C. Racial/ethnic inequality in the association of allostatic load and dental caries in children. J Public Health Dent. 2022;82(2): 239–46. doi: 10.1111/jphd.12470 34254682

[6] Gazzaz AZ, Carpiano RM, Laronde DM, Aleksejuniene J. Parental psychosocial factors, unmet dental needs and preventative dental care in children and adolescents with special health care needs: A stress process model. BMC Oral Health. 2022;22: 282. 10.1186/s12903-022-02314-y 35818050 PMC9275152

[7] Finlayson TL, Williams DR, Siefert K, Jackson JS, Nowjack-Raymer R. Oral health disparities and psychosocial correlates of self-rated oral health in the national survey of American life. Am J Public Health. 2010;100(S1): S246–55. doi: 10.2105/AJPH.2009.167783 20147685 PMC2837435

[8] Gomaa N, Glogauer M, Tenenbaum H, Siddiqi A, Quiñonez C. Social-biological interactions in oral disease: A ‘cells to society’ view. PLoS ONE. 2016;11(1): e0146218. doi: 10.1371/journal.pone.0146218 26751953 PMC4709106

[9] Gunepin M, Derache F, Trousselard M, Salsou B, Risso JJ. Impact of chronic stress on periodontal health. J Oral Med Oral Surg. 2018:24: 44–50. doi: 10.1051/mbcb/2017028

[10] Sabbah W, Gomaa N, Gireesh A. Stress, allostatic load, and periodontal diseases. Periodontol 2000. 2018;78: 154–161. doi: 10.1111/prd.12238 30198126

[11] World Health Organization. A conceptual framework for action on the social determinants of health. World Health Organization; 2010 [cited 2022 Apr 22]. Available from: https://apps.who.int/iris/handle/10665/44489

[12] Bourdieu P. The forms of capital. In Richardson, J. (ed) *Handbook of Theory and Research for the Sociology of Education*. New York: Greenwood; 1986.

[13] Coleman JS. Social capital in the creation of human capital. Am J Sociol. 1988;94: 95–120. 10.1086/228943

[14] Pinxten W, Lievens J. The importance of economic, social and cultural capital in understanding health inequalities: Using a Bourdieu-based approach in research on physical and mental health perceptions. Sociol Health Illn. 2014;36(7): 1095–110. doi: 10.1111/1467-9566.12154 25040507

[15] Song L. Social capital and psychological distress. J Health Soc Behav. 2011;52(4): 478–92. doi: 10.1177/0022146511411921 22021655

[16] Brennan DS, Spencer AJ. Life events and oral-health–related quality of life among young adults. Qual Life Res. 2009;18: 557–65. doi: 10.1007/s11136-009-9479-x 19404772

[17] Tsakos G, Sabbah W, Chandola T, Newton T, Kawachi I, Aida J, et al. Social relationships and oral health among adults aged 60 years or older. Psychosom Med. 2013;75(2): 178–86. doi: 10.1097/PSY.0b013e31827d221b 23324876

[18] Monteiro da Silva AM, Oakley DA, Newman HN, Nohl FS, Lloyd HM. Psychosocial factors and adult onset rapidly progressive periodontitis. J Clin Periodontol. 1996;23: 789–94. doi: 10.1111/j.1600-051x.1996.tb00611.x 8877667

[19] Merchant AT, Pitiphat W, Ahmed B, Kawachi I, Joshipura K. A prospective study of social support, anger expression and risk of periodontitis in men. J Am Dent Assoc. 2003;134: 1591–6. doi: 10.14219/jada.archive.2003.0104 14719755

[20] McGrath C, Bedi R. Influences of social support on the oral health of older people in Britain. J Oral Rehabil. 2002;29: 918–22. doi: 10.1046/j.1365-2842.2002.00931.x 12421323

[21] Sabbah W, Tsakos G, Chandola T, Newton T, Kawachi I, Sheiham A, et al. The relationship between social network, social support and periodontal disease among older Americans. J Clin Periodontol. 2011;38: 547–52. doi: 10.1111/j.1600-051X.2011.01713.x 21362014 PMC3091988

[22] Health Canada. Report on the findings of the Oral Health Component of the Canadian Health Measures Survey 2007–2009. Ottawa, Ontario: Ministry of Health, Government of Canada; 2010.

[23] Marcenes W, Kassebaum NJ, Bernabé E, Flaxman A, Naghavi M, Lopez A, et al. Global burden of oral conditions in 1990–2010: A systematic analysis. J Dent Res. 2013;92: 592–7. doi: 10.1177/0022034513490168 23720570 PMC4484374

[24] Ravaghi V, Quiñonez C, Allison PJ. The magnitude of oral health inequalities in Canada: Findings of the Canadian health measures survey. Community Dent Oral Epidemiol. 2013;41(6): 490–8. doi: 10.1111/cdoe.12043 23383978

[25] Guarnizo-Herreño CC, Watt RG, Fuller E, Steele JG, Shen J, Morris S, et al. Socioeconomic position and subjective oral health: Findings for the adult population in England, Wales and Northern Ireland. BMC Public Health. 2014;14: 827. doi: 10.1186/1471-2458-14-827 25107286 PMC4137102

[26] Fang C, Aldossri M, Farmer J, Gomaa N, Quiñonez C, Ravaghi V. Changes in income-related inequalities in oral health status in Ontario, Canada. Community Dent Oral Epidemiol. 2021;49: 110–8. doi: 10.1111/cdoe.12582 33044034

[27] Tsakos G, Demakakos P, Breeze E, Watt RG. (2011). Social gradients in oral health in older adults: Findings from the English Longitudinal Survey of Aging. Am J Public Health. 2011;101(10): 1892–9. doi: 10.2105/AJPH.2011.300215 21852627 PMC3222342

[28] Gomaa N, Nicolau B, Siddiqi A, Tenenbaum H, Glogauer M, Quiñonez C. How does the social “get under the gums”? The role of socio-economic position in the oral-systemic health link. Can J Public Health. 2017;108(3): e224–8. doi: 10.17269/CJPH.108.5930 28910242 PMC6972450

[29] Cohen S, Wills TA. Stress, social support, and the buffering hypothesis. Psychol Bull. 1985;98(2): 310–57. 10.1037/0033-2909.98.2.310 3901065

[30] Haag DG, Santiago PR, Schuch HS, Brennan DS, Jamieson LM. Is the association between social support and oral health modified by household income? Findings from a national study of adults in Australia. Community Dent Oral Epidemiol. 2022;50: 484–92. doi: 10.1111/cdoe.12693 34989422

[31] Statistics Canada. Canadian Community Health Survey: Combined Data, 2017 and 2018. Statistics Canada; 2019 [cited 2022 Apr 22]. Available from: https://www150.statcan.gc.ca/n1/daily-quotidien/191022/dq191022d-eng.htm.

[32] Littman A, White E, Satia J, Bowen D, Kristal A. Reliability and validity of 2 single-item measure of psychosocial stress. Epidemiol. 2006;17(4): 398–403. 10.1097/01.ede.0000219721.89552.51 16641618

[33] Statistics Canada. Appendix G: Estimated number of households and average household size by domain, Canada. *User Guide for the Survey of Household Spending*, *2017*. Statistics Canada; 2018 [cited 2022 Aug 1]. Available from https://www150.statcan.gc.ca/n1/pub/62f0026m/2018001/app-ann-g-eng.htm.

[34] Statistics Canada. Table 11-10-0241-01 Low income cut-offs (LICOs) before and after tax by community size and family size, in current dollars. Statistics Canada; 2022. 10.25318/1110024101-eng.

[35] Knol MJ, VanderWeele TJ. Recommendations for presenting analyses of effect modification and interaction. Int J Epidemiol. 2012;41(2): 514–20. doi: 10.1093/ije/dyr218 22253321 PMC3324457

[36] VanderWeele TJ. Reconsidering the denominator of the attributable proportion for interaction. Eur J Epidemiol. 2013;28: 779–84. doi: 10.1007/s10654-013-9843-6 24037116 PMC3882023

[37] Armfield JM, Mejía GC, Jamieson LM. Socioeconomic and psychosocial correlates of oral health. Int Dent J. 2013;63(4): 202–9. doi: 10.1111/idj.12032 23879256 PMC9375003

[38] Griffin SO, Jones JA, Brunson D, Griffin PM, Bailey WD. Burden of oral disease among older adults and implications for public health priorities. Am J Public Health. 2012;102:411–418. doi: 10.2105/AJPH.2011.300362 22390504 PMC3487659

[39] Patel J, Wallace J, Doshi M, Gadanya M, Yahya IB, Roseman J, et al. Oral health for healthy ageing. The Lancet Healthy Longevity. 2021;2(8):e521–e527. doi: 10.1016/S2666-7568(21)00142-2 36098001

[40] Manski RJ, Moeller J, Chen H, St. Clair PA, Schimmel J, Magder L, et al. Dental care utilization and retirement. J Pub Health Dent. 2010;70:67–75. doi: 10.1111/j.1752-7325.2009.00145.x 19765203 PMC2864359

[41] Courtin E, Knapp M. Social isolation, loneliness and health in old age: A scoping review. Health and Social Care in the Community. 2017;25(3):799–812. doi: 10.1111/hsc.12311 26712585

[42] Cornwell B, Laumann EO, Schumm LP. The Social Connectedness of Older Adults: A National Profile. Am Sociol Review. 2008;73:185–203. doi: 10.1177/000312240807300201 19018292 PMC2583428

[43] McDonald S, Mair CA. Social capital across the life course: Age and gendered patterns of network resources. Sociological Forum. 2010;25:335–359. 10.1111/j.1573-7861.2010.01179.x

[44] Rouxel P, Tsakos G, Demakakos P, Zaninotto P, Chandola T, Watt RG. Is social capital a determinant of oral health among older adults? Findings from the English longitudinal study of ageing. PloS ONE. 2015;10(5): e0125557. doi: 10.1371/journal.pone.0125557 25992569 PMC4436243

[45] Moore S, Kawachi I. Twenty years of social capital and health research: a glossary. J Epidemiol Community Health. 2017;71(5): 513–7. doi: 10.1136/jech-2016-208313 28087811

[46] Vettore MV, Abreu MHNG, Mendes SR, e E. Do changes in income and social networks influence self-rated oral health trajectories among civil servants in Brazil? Evidence from the longitudinal Pró-Saúde study. BMC Oral Health. 2022;22: 153. 10.1186/s12903-022-02191-5 35488334 PMC9052516

[47] Prior L, Manley D, Jones K. Stressed out? An investigation of whether allostatic load mediates associations between neighbourhood deprivation and health. Health & Place. 2018;52: 25–33. doi: 10.1016/j.healthplace.2018.05.003 29775832

[48] Seeman TE, Singer BH, Rowe JW, Horwitz RI, McEwen BS. Price of adaptation–allostatic load and its health consequences. Arch Intern Med. 1997;157(19): 2259–68. 10.1001/archinte.1997.00440400111013 9343003

[49] Porcelli P, Laera D, Mastrangelo D, Di Masi A. Prevalence of allostatic overload syndrome in patients with chronic cardiovascular disease. Psychother Psychosom. 2012;81(6): 375–7. doi: 10.1159/000341179 22964661

[50] Scheuer S, Wiggert N, Brückl TM, Awaloff Y, Uhr M, Lucae S, et al. Childhood abuse and depression in adulthood: The mediating role of allostatic load. Psychoneuroendocr. 2018;94: 134–42. doi: 10.1016/j.psyneuen.2018.04.020 29775876

[51] Honkalampi K, Virtanen M, Hinsta T, Ruusunen A, Mäntyselkä P, Ali-Sisto T, et al. Comparison of the level of allostatic load between patients with major depression and the general population. J Psychosom Res. 2021;143: e110389. doi: 10.1016/j.jpsychores.2021.110389 33609985

[52] Vasiliou A, Shankardass K, Nisenbaum R, Quiñonez C. Current stress and poor oral health. BMC Oral Health. 2016;16: 88. doi: 10.1186/s12903-016-0284-y 27590184 PMC5010733

[53] Khemka S, Baliga S, Thosar N. Approaches to improve access to dental care services. Int Dent Med J Adv Res. 2015;1: 1–4.

[54] Abegunde DO, Mather CD, Adam T, Ortegon M, Strong K. The burden and costs of chronic diseases in low-income and middle-income countries. The Lancet. 2007;370(9603): 8–14. doi: 10.1016/S0140-6736(07)61696-1 18063029

[55] Ahnquist J, Wamala SP, Lindstrom M. Social determinants of health–A question of social or economic capital? Interaction effects of socioeconomic factors on health outcomes. Soc Sci Med. 2012;74(6): 930–9. 10.1016/j.socscimed.2011.11.026 22305807

[56] Parbhakar KK, Farmer JW, Quiñonez C. The influence of living conditions and individual behaviours on the oral-systemic disease connection: A cross-sectional analysis. J Public Health Dent. 2021;82(2): 220–8. doi: 10.1111/jphd.12455 33890301

[57] Celeste RK, Nadanovsky P. How much of the income inequality effect can be explained by public policy? Evidence from oral health in Brazil. Health Policy. 2010;97(2–3): 250–8. doi: 10.1016/j.healthpol.2010.05.015 20557971

[58] Peres MA, Macpherson LMD, Weyant RJ, Daly B, Venturelli R, Mathur MR, et al. Oral diseases: A global public health challenge. The Lancet. 2019;394(10194): 249–60. doi: 10.1016/S0140-6736(19)31146-8 31327369

[59] Guarnizo-Herreño CC, Scholes S, Heilmann A, O’Connor R, Fuller E, Shen J, et al. Dental attendance and behavioural pathways to adult oral health inequalities. J Epidemiol Community Health. 2021;75: 1063–9. doi: 10.1136/jech-2020-216072 33893184

